# *IGHMBP2* mutation associated with organ-specific autonomic dysfunction

**DOI:** 10.1016/j.nmd.2018.08.010

**Published:** 2018-12

**Authors:** Pedro J. Tomaselli, Alejandro Horga, Alexander M. Rossor, Zane Jaunmuktane, Andrea Cortese, Julian C. Blake, Natalia Zarate-Lopez, Henry Houlden, Mary M. Reilly

**Affiliations:** a MRC Centre for Neuromuscular Diseases, National Hospital for Neurology and Neurosurgery and UCL Institute of Neurology, Queen Square, London WC1N 3AR, UK; b Department of Neuromuscular Disorders, Clinical Hospital of Ribeirão Preto, University of São Paulo, Ribeirão Preto, SP 14640-900, Brazil; c Division of Neuropathology, National Hospital for Neurology and Neurosurgery and UCL Institute of Neurology, Queen Square, London WC1N 3AR, UK; d Department of Clinical Neurophysiology, Norfolk and Norwich University Hospital, Norwich NR4 7UY, UK; e Department of Gastroenterology, University College London Hospitals, London NW1 2BU, UK

**Keywords:** CMT, IGHMBP2 gene, Target multigene panel, Next generation sequencing, SMARD1

## Abstract

Biallelic mutations in the *IGHMBP*2 have been associated with two distinct phenotypes: spinal muscular atrophy with respiratory distress type 1 (SMARD1) and CMT2S. We describe a patient who developed progressive muscle weakness and wasting in her upper and lower limbs from infancy. She developed respiratory involvement at age 9, eventually requiring 24-h non-invasive ventilation, and severe autonomic dysfunction restricted to the gastrointestinal tract. Neurophysiological studies at age 27 years revealed absent sensory and motor responses and severe chronic denervation changes in proximal muscles of the upper limbs. Targeted multigene panel sequencing detected a novel homozygous missense variant in the *IGHMBP2* gene (c.1325A > G; p.Tyr442Cys). This variant was validated by Sanger sequencing and co-segregation analysis confirmed that both parents were asymptomatic heterozygous carriers. This case report confirms that IGHMBP2 related disorders can result in a severe peripheral neuropathy with gastrointestinal autonomic dysfunction requiring parenteral nutrition.

## Highlights

• Novel IGHMBP2 variant found in a patient with early onset severe peripheral neuropathy.
• IGHMBP2 mutations may cause enteral autonomic dysfunction.
• Autonomic dysfunction in IGHMBP2-related disorders may be severe requiring parenteral nutrition.

## 1 Introduction

Autosomal recessive mutations in the immunoglobulin μ-binding protein 2 gene (*IGHMBP2*) have been associated with two distinct phenotypes: spinal muscle atrophy with respiratory distress type 1 (SMARD1) and, more recently, Charcot-Marie-Tooth disease type 2S (CMT2S). In SMARD1, degeneration of alpha motor neurons of the spinal cord results in diaphragmatic paralysis and distal muscular atrophy. Sensory and autonomic nerves are also affected in some patients. The disorder usually starts in infancy and early death due to respiratory distress is a hallmark of the disease, although patients with milder phenotypes (later onset and survival until 15–20 years of age) have been described [1], [2]. In contrast, patients with CMT2S develop an axonal neuropathy manifesting with slowly progressive, distal-predominant muscle weakness and sensory loss with preserved respiratory function [3], [4].

We describe a case of motor and sensory axonal neuropathy associated with respiratory involvement and severe gastrointestinal organ-specific autonomic dysfunction due to a novel missense mutation in *IGHMBP2*, demonstrating that IGHMBP2 related disorders can result in a severe peripheral neuropathy with enteral autonomic dysfunction.

## 2 Case report

The patient was evaluated in the inherited neuropathy clinic in the Centre for Neuromuscular Diseases, National Hospital for Neurology and Neurosurgery, London, UK. Written informed consent was obtained for participation in the study and for publication of images. Methods for whole exome sequencing (WES), targeted multigene panel and Sanger sequencing are provided in the Supplementary Material. Nerve conduction studies, EMG and nerve biopsy were performed using standard methods. This study was approved by the local research ethics committee.

### 2.1 Clinical history and phenotype

The patient was the eldest daughter of two siblings born to consanguineous healthy parents of Iranian origin. Her family history was unremarkable. She presented with hypotonia and delayed walking during infancy. Despite this, she was able to walk independently until 4 years of age, when she started to use ankle-foot orthoses. By 6 years of age, she was wheelchair-bound. Following an episode of pneumonia at 9 years of age, she was started on nocturnal non-invasive ventilation and over the subsequent months, she became 24 h ventilator dependent. She reported no sensory or urinary symptoms, abnormal sweating or vasomotor symptoms.

She developed gastrointestinal symptoms in the first decade of life characterized by abdominal bloating, infrequent bowel movements and constipation. Additional gastrointestinal symptoms developed over the following years, including excessive saliva, dysphagia and odynophagia eventually resulting in intermittent nasogastric tube placement to relieve the gastric gaseous distension.

Unfortunately, her gastrointestinal symptoms gradually worsened and, at age 14 years, she developed recurrent episodes of acute bowel distension affecting her respiratory capacity and requiring more frequent nasogastric tube placement. At age 27 years, a percutaneous endoscopic gastrostomy (PEG) was performed from which 1–3 l of gastric content was drained per day because of gastroparesis and slow bowel function.

At age 28 years, oesophageal intraluminal impedance testing showed excessive gastroesophageal reflux. Anorectal physiology studies confirmed normal sphincter function and rectal sensation but despite this, her constipation worsened to the extent she required an enema every other day. Prokinetic medications had limited effect on her symptoms and her weight decreased. At age 30 years, she was started on total parenteral nutrition.

**Fig. 1:**
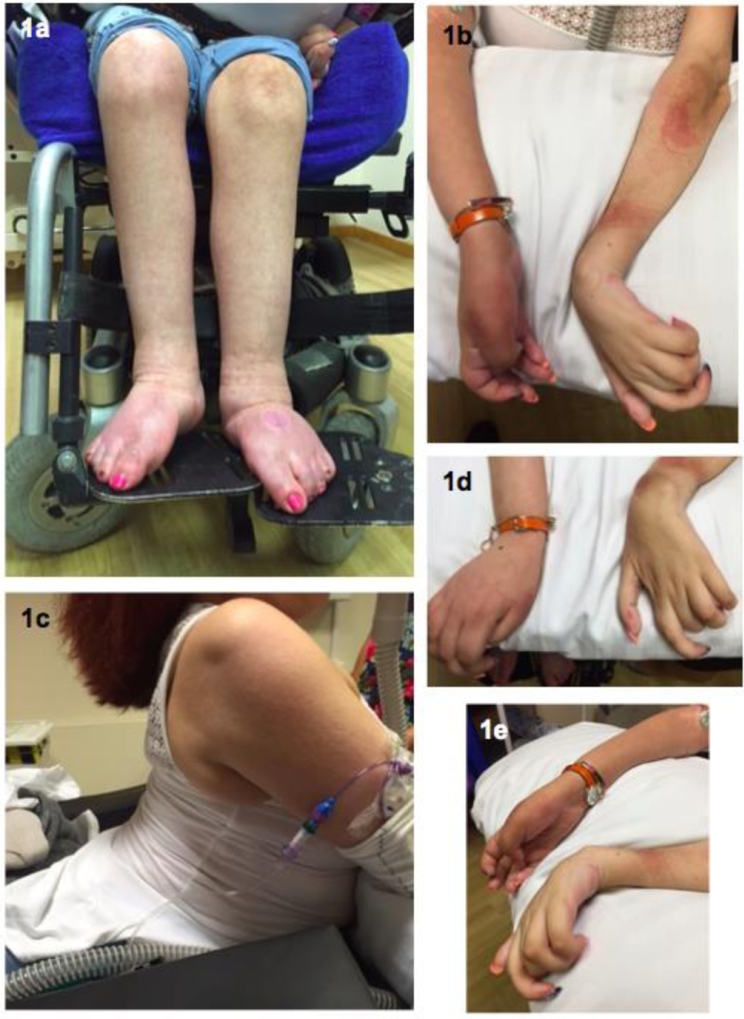
Clinical images of the proband. Photographs of the patient's legs, arms and trunk show severe wasting distally and proximally in upper and lower limbs and severe kyphoscoliosis.

Examination at 27 years of age revealed severe kyphoscoliosis (Fig. 1). Cranial nerve examination, including eye movements, tongue and palate, was normal. There was mild neck flexion weakness. Her upper and lower limbs were wasted proximally and distally and she was severely weak globally with Medical Research Council (MRC) grades for shoulder adduction 4 + /5, elbow flexion and extension 1/5 and no movement of the intrinsic hand muscles or in the lower limbs. Deep tendon reflexes were absent and plantar responses were mute. Pinprick sensation was normal throughout. Vibration sense was absent at the toes and ankles and proprioception was absent at the toes.

Subsequent examinations confirmed progression of the motor and sensory deficits. Nerve conduction studies performed at age 27 years revealed absent sensory and motor responses throughout. Needle EMG showed severe chronic denervation in proximal muscles of the upper limbs (biceps and deltoid).

### 2.2 Genetics studies

Targeted Sanger sequencing of *MFN2, MPZ, LMNA, MT-ATP6* and *MT-ATP8* genes revealed no pathogenic mutations. Given the atypical phenotype, we performed whole exome sequencing (WES) which revealed a novel heterozygous variant in IGHMBP2 (c.1325A > G; p.Tyr442Cys). WES coverage of some genes involved in hereditary neuropathy was low (Supplementary Table 1) and we therefore decided to proceed with targeted multi-gene panel testing to confirm the absence of mutations in common neuropathy-related genes. Interestingly, the targeted multi-gene panel detected the same variant in *IGHMBP2* detected by WES but in homozygosity (see Supplementary Table 2 for genes included and alignment statistics). *In silico* analyses revealed that, this variant involved an evolutionarily conserved amino acid, and was predicted to be pathogenic (Supplementary Material). The homozygous variant was validated by Sanger sequencing and the analysis of parental DNA samples confirmed that both parents were heterozygous carriers.

### 2.3 Nerve biopsy at age 3

**Fig. 2:**
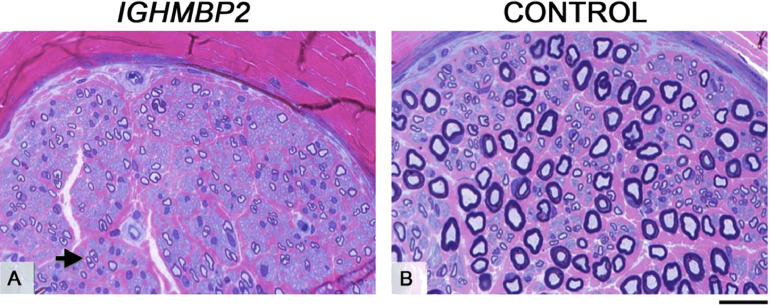
Sural nerve biopsy. Semi-thin resin sections from the patient's sural nerve (A) and age-matched control (B), stained with methylene blue azure basic fuchsin (MBA-BF). The nerve fascicles in the patient's biopsy in comparison with the control were of very small size. Image A shows reduced fibre density, small fibre size and complete absence of large normally myelinated fibres across the fascicles. There was no evidence of active axonal degeneration or signs of demyelinating and remyelinating process.There were occasional clusters of regeneration (A, arrow). Scale bar (A and B): 25 μm.

All fascicles in the biopsy were small. Nerve fibres across the fascicles were small and moderately thinly myelinated. There was no evidence of ongoing active axonal degeneration or demyelination and no inflammatory infiltrates were observed (Fig. 2). Teased fibre preparation showed uniformly short internodes suggesting some regeneration. Electronic microscopy revealed some myelinated fibres with reduplicated basement membranes and a few small clusters of regeneration.

## 3 Discussion

We describe a patient with childhood onset autosomal recessive motor and sensory neuropathy with severe organ-specific autonomic dysfunction due to a novel *IGHMBP2* missense variant. The mutation was miscalled as a heterozygous allele on whole exome sequencing due to poor coverage but was detected in homozygosity using a targeted gene panel.

The present case has features that diverge from classical SMARD1 and CMT2S phenotypes. In contrast to SMARD1, in which most patients require assisted ventilation within the first year of life and die within the first decade of life, our patient developed respiratory involvement requiring non-invasive ventilation at age 9 years and is still alive in her 30 s. Sensory involvement and autonomic dysfunction, including excessive sweating, tachycardia, constipation and bladder dysfunction, have been described in patients with SMARD1 but also in patients with CMT2S due to IGHMBP2 mutations. Our patient's phenotype is therefore more typical of a severe CMT2S phenotype within the spectrum of *IGHMBP2* related disorders.

It is not clear why our patient has autonomic dysfunction restricted to the gastrointestinal tract. To our knowledge, there have been no studies of the enteric plexus in the NMD mouse or in post mortem tissue of patients with SMARD1 or CMT2S. Autonomic involvement in patients with *IGHMBP2*-related disorders has been previously described. Jedrzejowska et al. reported five SMARD1 patients from four different families with autonomic dysfunction, including mild-to-severe excessive sweating, tachycardia, constipation and bladder dysfunction [5]. Schottmann et al. reported two siblings with CMT2S and progressive autonomic features including gastrointestinal and bladder dysfunction in association with an axonal sensorimotor neuropathy without respiratory distress, one of whom required surgery for achalasia at age 18 years [4]. It's possible that the patient has more widespread autonomic dysfunction, however, due to the patient's severe disability we were unable to undertake more extensive autonomic function testing.

*IGHMBP2* encodes a protein belonging to the putative helicase superfamily. It is expressed in developing and adult human brain, with high expression in the cerebellum [3]. It is functionally linked to translation and may be a component of the translational machinery [6], [7].

We suggest that mutations in *IGHMBP2* may cause an early onset severe sensory motor axonal neuropathy in association with severe organ specific autonomic neuropathy affecting the gastrointestinal tract.

## Websites

Exome Variant Server: http://evs.gs.washington.edu/EVS. Accessed May 12, 2017.

Exome Aggregation Consortium: http://exac.broadinstitute.org. Accessed May 12, 2017.

Inherited Neuropathy Variant Browser: http://hihg.med.miami.edu/code/http/cmt/public_html/index.html#/.

## Appendix Supplementary materials

Image, application 1
