# Supplementary material for: IGHMBP2 mutation associated with organ-specific autonomic dysfunction
Source: Neuromuscul Disord. 2018 Dec;28(12):1012–5. doi: 10.1016/j.nmd.2018.08.010 (PMC6302219; doi:10.1016/j.nmd.2018.08.010)
Supplement: Supplementary file 1 [file mmc1.pdf]

Supplementary Material

Next generation sequencing

WES and targeted multigene panel on DNA from blood sample obtained from patient was performed at Neurogenetics Laboratory of UCL Institute of Neurology; Queen Square, London, UK using the Illumina's Nextera rapid capture focused enrichment kit and run on the Illumina HiSeq 2500 instrument. The resulting 100bp paired-end sequence reads were mapped against the human reference genome assembly 19 (GRCh37) with the Burrows-Wheeler Aligner (BWA) package. NGS analysis was performed using in house pipeline.

Whole exome sequencing

WES revealed a total of 22,346 exonic variants. Variants were filtered for non-synonymous, splice-site, and coding indel variants that had a minor allele frequency (MAF) <0.1% in the Exome Variant Server (EVS), 1000 genome project and Exome Aggregation Consortium database (ExAC). 323 variants met these filtering criteria, and one variant was in a gene associated with inherited neuropathies. The c.1325A>G; p.Tyr442Cys, was found in heterozygosity in *IGHMBP2*. This variant has not been previously reported in the literature or to public databases of genetic variation (dbSNP, NHLBI exome variant server, ExAC database). It is located in a high conserved region (phastCons = 1 and phyloP = 4.56), there is a large physiochemical difference between Tyrosin (Tyr) and Cysteine (Cys) and has been predicted to be pathogenic by SIFT (1), MutationTaster (p-value: 1), and Polyphen2 (1).

Targeted multigene panel

Target multigene panel analysis revealed that 99.82% of the coding exons (+/- base pairs, except GJB1, for which the target region is extended 860 bases upstream of the ATG start codon to include the nerve specific promoter region) of all genes in the panel were sequenced to a read depth of 30X or greater. The minimum coverage depth of coverage was 19X for AARS gene. The regions with coverage bellow 30X were analyzed manually.

Sanger sequencing

PCRs were performed with AmpliTaq Gold 360 Master Mix (Applied Biosystems, Foster City, California, USA), with an annealing temperature of 58ºC. Sequencing reactions were performed using M13 primers and BigDye Terminator cycle sequencing chemistry (Applied Biosystems, Foster City, California, USA). Sequences were analyzed on an ABI3730XL automated DNA sequencer and SeqScape software (Applied Biosystems, Foster City, California, USA). Primers are available under request.

Supplementary table 1. List of genes related to CMT2 and hereditary motor neuropathy and alignment statistics of depth coverage on WES.

	Gene	On-target					
		≥1X (%)	≥10 (%)	≥30X (%)	Average depth	Median depth	Max depth
**Alanyl-tRNA synthetase**	**AARS**	100	98	96	99	97	192
**ATPase Cu$^{(2+)}$-transporting, alpha polypeptide**	**ATP7A**	100	100	88	72	68	185
**Bicaudal D homolog of drosophila 2**	**BICD2**	100	84	54	39	34	114
**BSCL2**	**BSCL2**	98	94	87	88	94	182
**Dynactin 1**	**DCTN1**	100	100	87	88	88	194
**Dynamin 2**	**DNM2**	100	100	90	94	97	247
**Dynein, cytoplasmic 1, heavy chain 1**	**DYNC1H1**	100	100	96	101	100	218
**Early growth response 2**	**EGR2**	100	74	58	56	45	189
**FGD1-related F-actin binding protein**	**FGD4**	100	99	91	68	70	134
**SAC domain containing inositol phosphatase 3**	**FIG4**	100	100	97	77	72	180
**Glycyl-tRNA syntheyase**	**GARS**	100	100	94	88	88	207
**Ganglioside-induved differentiation-associated protein 1**	**GDAP1**	100	100	100	104	98	208
**GAP junction protein, beta-1**	**GJB1 (including promoter region)**	67	48	32	24	29	115
**Histidine triad nucleotide-binding protein 1**	**HINT1**	100	91	65	47	45	112
**Heat-shock 27-KD protein 1**	**HSPB1**	100	84	77	85	85	197
**Heat-shock 27-KD protein 8**	**HSPB8**	100	79	73	79	84	182
**Immunoglobulin mu-binding protein 2**	**IGHMBP2**	100	97	85	71	69	177
**LPS-induced TNF-alpha factor**	**LITAF**	100	91	68	52	54	130
**Lamin A/C**	**LMNA**	99	94	84	76	71	186

**Leucin-rich repeat and sterile alpha motif containing 1**	**LRSAM1**	100	100	96	98	99	204
**Methionyl-tRNA synthetase**	**MARS**	100	93	63	44	41	140
**Mitofusin 2**	**MFN2**	100	100	97	101	101	215
**Myelin protein zero**	**MPZ**	100	100	76	61	57	150
**Myotubularin-related protein 2**	**MTMR2**	100	100	100	90	88	183
**NMYC downstream-regulated gene 1**	**NDRG1**	100	90	78	67	67	146
**Neurofilament protein, light polypeptide**	**NEFL**	100	96	78	60	57	146
**Pheripheral myelin protein 22**	**PMP22**	100	100	100	104	101	171
**Phosphoribosylpyrophosphate synthetase 1**	**PRPS1**	100	100	100	95	90	170
**Periaxin**	**PRX**	100	92	75	75	63	257
**Sterile alpha motif domain-containing protein 9**	**RAB7A**	100	94	92	94	104	142
**SET-binding factor 2**	**SBF2**	100	100	95	73	73	159
**Senataxin**	**SETX**	100	98	83	72	67	238
**SH3 domain and tetratricopeptide repeat domain 2**	**SH3TC2**	100	98	87	82	83	219
**Solute carrier family 52 (Riboflavin transporter), member 1**	**SLC52A1**	98	72	41	28	25	92
**Solute carrier family 52 (Riboflavin transporter), member 2**	**SLC52A2**	100	83	50	47	31	175
**Solute carrier family 52 (Riboflavin transporter), member 3**	**SLC52A3**	100	91	76	68	60	208
**Transient receptor potential cation channel, subfamily 5, member 4**	**TRPV4**	100	93	78	69	67	210
**Valosin containing protein**	**VCP**	100	98	95	108	108	231
**Tyrosyl-tRNA synthetase**	**YARS**	100	100	95	88	86	239

Supplementary table 2. List of genes related to CMT2 and hereditary motor neuropathy and alignment statistics of depth coverage on targeted multigene panel.

**Gene**		**≥1X (%)**	**≥10X (%)**	**≥30X (%)**	**Average depth**	**Median depth**	**Max depth**
**Alanyl-tRNA synthetase**	**AARS**	100	100	99.6	328	322	756
**BSCL2**	**BSCL2**	100	100	100	303	263	779
**Dynamin 2**	**DNM2**	100	100	100	288	280	781
**Dynein, cytoplasmic 1, heavy chain 1**	**DYNC1H1**	100	100	99.9	358	329	963
**Glycyl-tRNA syntheyase**	**GARS**	100	100	99.8	273	240	741
**Ganglioside-induved differentiation-associated protein 1**	**GDAP1**	100	100	100	472	387	1170
**GAP junction protein, beta-1**	**GJB1 (Including promoter region)**	100	100	100	329	274	601
**Histidine triad nucleotide-binding protein 1**	**HINT1**	100	100	100	405	363	816
**Heat-shock 27-KD protein 1**	**HSPB1**	100	100	100	366	299	656
**Heat-shock 27-KD protein 8**	**HSPB8**	100	100	100	303	283	572
**Immunoglobulin mu-binding protein 2**	**IGHMBP2**	100	100	100	334	333	702
**Lamin A/C**	**LMNA**	100	100	100	374	352	949
**Leucin-rich repeat and sterile alpha motif containing 1**	**LRSAM1**	100	100	98.2	276	246	784
**Methionyl-tRNA synthetase**	**MARS**	100	100	100	368	359	704
**Mitofusin 2**	**MFN2**	100	100	100	281	278	695
**Myelin protein zero**	**MPZ**	100	100	100	327	292	801
**Neurofilament protein, light polypeptide**	**NEFL**	100	100	100	292	223	689
**Pheripheral myelin protein 22**	**PMP22**	100	100	100	546	545	1044
**Phosphoribosylpyrophosphate synthetase 1**	**PRPS1**	100	100	100	372	354	645
**Sterile alpha motif domain-containing protein 9**	**RAB7A**	100	100	100	456	395	884
**SH3 domain and tetratricopeptide repeat domain 2**	**SH3TC2**	100	100	100	357	381	508
**Transient receptor potential cation channel, subfamily 5, member 4**	**TRPV4**	100	100	100	362	377	676

**Valosin containing protein**	**VCP**	100	100	98.67	310	278	776
**Tyrosyl-tRNA synthetase**	**YARS**	100	100	100	315	322	640
